# p38α Signaling Is Associated with Pancreatic Ductal Adenocarcinoma Progression and Represents a Potential Therapeutic Vulnerability

**DOI:** 10.3390/ijms27198550

**Published:** 2026-09-24

**Authors:** Ilaria Grassi, Elisabetta Di Nicola, Erica Candela, Elisabetta Cavalcanti, Annamaria Valentini, Leonardo Vincenti, Carla Santoiemma, Valentina Grossi, Martina Lepore Signorile, Cristiano Simone, Raffaele Armentano

**Affiliations:** 1 Histopathology Unit, National Institute of Gastroenterology, IRCCS “Saverio de Bellis” Research Hospital, Castellana Grotte, 70013 Bari, Italy; ilaria.grassi@irccsdebellis.it (I.G.); elisabetta.cavalcanti@irccsdebellis.it (E.C.); am.valentini@irccsdebellis.it (A.V.); raffaele.armentano@irccsdebellis.it (R.A.); 2 Medical Genetics, National Institute of Gastroenterology, IRCCS “Saverio de Bellis” Research Hospital, Castellana Grotte, 70013 Bari, Italy; elisabetta.dinicola@irccsdebellis.it or elisabetta.dinicola@uniroma1.it (E.D.N.); erica.candela@irccsdebellis.it (E.C.); carla.santoiemma@irccsdebellis.it (C.S.); valentina.grossi@irccsdebellis.it (V.G.); 3 Department of Molecular Medicine, University of Rome La Sapienza, 00161 Rome, Italy; 4 Department of General Surgery, National Institute of Gastroenterology, IRCCS “Saverio de Bellis” Research Hospital, Castellana Grotte, 70013 Bari, Italy; leonardo.vincenti@irccsdebellis.it; 5 Department of Precision and Regenerative Medicine and Jonic Area (DiMePRe-J), University of Bari Aldo Moro, 70124 Bari, Italy

**Keywords:** pancreatic ductal adenocarcinoma, intraductal papillary mucinous neoplasms, p38α, patient-derived tumor organoids

## Abstract

Pancreatic ductal adenocarcinoma (PDAC) is one of the most lethal malignancies, and approximately 15% of cases arise from intraductal papillary mucinous neoplasms (IPMNs). These precursor lesions can progress to invasive cancer through the accumulation of molecular alterations. To identify pathways associated with this transition, we performed comparative transcriptomic analyses of patient-derived IPMN and PDAC samples, followed by protein-level validation in tissue specimens and pancreatic cancer cell lines. Functional studies were conducted using the selective p38α inhibitor ralimetinib in two-dimensional cultures, three-dimensional tumorspheres, and patient-derived tumor organoids (PDTOs). Transcriptomic profiling revealed significant downregulation of the p38 pathway in IPMNs compared with PDAC, suggesting progressive pathway activation during pancreatic tumorigenesis. Immunohistochemical analyses confirmed increased phospho-activated p38α in PDAC tissues, particularly in lesions with advanced malignant features, while PDAC cell lines displayed higher levels of activated p38α than non-tumorigenic pancreatic epithelial cells. Pharmacological inhibition of p38α suppressed downstream MAPKAPK2 phosphorylation, induced apoptotic cell death, and reduced tumorsphere growth and viability. Consistently, PDTOs exhibiting elevated p38α activation showed increased sensitivity to ralimetinib treatment. Collectively, these findings identify p38α signaling as a pathway activated during PDAC progression and support further investigation of p38α-targeted therapies as a potential precision medicine strategy for pancreatic cancer.

## 1. Introduction

Pancreatic ductal adenocarcinoma (PDAC) is the most common histological subtype of pancreatic cancer, accounting for approximately 90% of all cases, and remains one of the leading causes of cancer-related mortality worldwide, with a five-year survival rate of only ~10% [1,2]. Its poor prognosis is largely driven by the absence of effective early-detection strategies, resulting in diagnosis at advanced stages of disease; indeed, more than 80% of patients are diagnosed with locally advanced or metastatic tumors at the time of diagnosis [3,4,5]. Consequently, only a minority of patients are eligible for surgical resection, which remains the only curative treatment for PC [6,7]. These observations underscore the urgent need for improved early-detection approaches and molecular classification of PDAC, in order to increase the proportion of patients eligible for curative treatment.

In terms of its origin, the majority of PDACs arise de novo from the pancreatic duct, while approximately 15% develop from precursor lesions known as intraductal papillary mucinous neoplasms (IPMNs) [8]. IPMNs are mucin-producing epithelial pancreatic cysts (>5 mm) classified by their anatomical location (main duct, branch duct, or mixed), histologic subtype (gastric, intestinal, or pancreatobiliary), and degree of dysplasia (low-grade vs. high-grade) [6,7,9,10]. These lesions are frequently detected incidentally during magnetic resonance imaging (MRI) or computed tomography (CT) scans performed for unrelated indications [11]. Despite advances in molecular characterization, histopathological examination remains the diagnostic gold standard [12,13], as no surrogate molecular markers have yet demonstrated sufficient accuracy and validation to replace direct morphological assessment.

The acquisition of invasive potential by these precursor lesions is a highly complex biological process involving progressive morphological and molecular alterations [14,15,16]. Consistent with this model, the genomic landscape of PDAC is characterized by the stepwise accumulation of genetic alterations at distinct stages of tumorigenesis, supporting a multistep model of tumor progression from normal pancreatic epithelium to IPMN and ultimately to invasive carcinoma [1,17]. Activating mutations in the KRAS oncogene are detected in more than 90% of patients with PDAC [18,19] and are also present in the majority of IPMNs, suggesting that KRAS alteration likely represents the earliest genetic event that drives both the initiation and progression of this malignancy [20,21]. Subsequently, alterations in TP53, SMAD4, and CDKN2A contribute to the malignant transformation of IPMNs into invasive PDAC [22,23,24]. The identification of biomarkers and signaling pathways associated with this transition is essential for improving risk stratification and for developing targeted strategies aimed at preventing PDAC progression. Therefore, it is imperative to further elucidate the genetic landscape underlying PDAC progression and metastasis.

Multiple studies have demonstrated that, in PDAC, oncogenic KRAS primarily exerts its tumor-promoting effects through activation of the mitogen-activated protein kinase (MAPK) signaling pathway [25]. The MAPK family comprises a highly conserved group of serine/threonine kinases that regulate diverse cellular processes, including proliferation, differentiation, survival, and tumor progression [26]. Among MAPK family members, the p38 subfamily has emerged as a critical mediator of cancer-associated signaling and consists of four isoforms: p38α (MAPK14), p38β (MAPK11), p38γ (MAPK12/SAPK3/ERK6), and p38δ (MAPK13) [27]. Accumulating evidence indicates that p38α and p38β promote cell proliferation, migration, and invasion in multiple tumor types, including PDAC, supporting their pro-tumorigenic role [28]. Activation of the p38α signaling cascade occurs through MKK4/6-dependent dual phosphorylation of p38α at Thr180/Tyr182, which subsequently phosphorylates and activates the downstream kinase MAPKAPK2 [29]. Notably, p38α expression is significantly upregulated in PDAC tissues and has been associated with increased tumor aggressiveness and poorer overall survival [30]. These findings, together with the observed functional dependency and pharmacological sensitivity of PDAC cells to p38α inhibition, support a potential role for p38α as a therapeutic target in PDAC. In this study, we performed comparative molecular analyses of patient-derived IPMN and PDAC specimens to identify factors associated with disease progression and to uncover potential therapeutic targets. In particular, we compared p38α expression to investigate its involvement in disease progression. Furthermore, we evaluated the antitumor effects of ralimetinib, a potent and selective inhibitor of p38α that competitively binds the ATP-binding pocket and suppresses kinase activity [31,32,33,34].

## 2. Results

### 2.1. Transcriptomic Profiling Reveals Enrichment of the p38 Pathway in PDAC Compared with IPMN

To investigate the molecular alterations associated with the progression from precursor lesions to invasive pancreatic cancer, we performed a cross-sectional study in which a total of 30 patients were included in the transcriptomic analysis, comprising 14 patients with IPMNs and 16 patients with PDAC. Table 1 summarizes the clinicopathological characteristics of the study population. Specifically, RNA sequencing (RNA-seq) analysis was performed on surgically resected IPMN and PDAC specimens. Gene set enrichment analysis (GSEA) identified significant transcriptomic differences between the two groups, with the p38 MAPK cascade (GO:0038066) emerging as one of the most significantly enriched pathways in PDAC compared with IPMN (Figure 1). Notably, the pathway showed an enrichment score (ES) of −0.477 and a normalized enrichment score (NES) of −2.65, with a nominal *p* value of 4.31 × 10^−9^, an adjusted *p* value of 6.60 × 10^−7^, and an FDR q-value of 4.48 × 10^−7^. Thus, enrichment of the p38 MAPK cascade remained statistically significant after correction for multiple testing. Given the well-established role of p38 signaling in regulating cellular stress responses, inflammation, cell survival, and tumor progression, these findings suggest that increased enrichment of this pathway is associated with the invasive PDAC phenotype.

### 2.2. PDAC Tissues and High-Grade Lesions Display an Increased Phosphorylation of p38α

To validate transcriptomic findings at the protein level, we assessed the activated phosphorylated form of p38α (p-p38α) by immunohistochemistry on formalin-fixed paraffin-embedded IPMN and PDAC tissue sections following hematoxylin and eosin histopathological evaluation. p-p38α positivity was observed in all patients with PDAC. Conversely, expression of p-p38α was absent in all patients with IPMN (Figure 2). Therefore, immunohistochemical analysis demonstrated a clear increase in p-p38α staining in PDAC tissues compared with IPMN lesions. Notably, phosphorylation levels were high in samples displaying advanced or malignant histopathological features (Table 1), supporting an association between increased p38α activation and the invasive PDAC phenotype. These findings are consistent with previous reports linking p38 activation to tumor aggressiveness, metastatic potential, and poor prognosis in pancreatic cancer [30] (Figure 2).

### 2.3. High p38α Expression Is Associated with a Trend Toward Worse Disease Outcomes

To further investigate the clinical significance of p38*α* expression in invasive pancreatic cancer, we performed an analysis of the TCGA-PAAD cohort (178 PAAD patients). To explore the clinical relevance of p38*α* expression, patients from the TCGA-PAAD cohort were stratified into p38*α*-high and p38*α*-low groups according to MAPK14 gene expression levels. Kaplan–Meier survival analysis revealed a trend toward reduced overall survival in patients with high p38*α* expression compared with those with low expression, although the difference did not reach statistical significance (log-rank *p* = 0.053) (Figure 3a).

Patients with high p38*α* expression consistently showed a higher frequency of adverse clinical features, including a trend toward reduced overall survival, increased occurrence of new neoplastic events after initial therapy, persistent tumor status, and metastatic disease, suggesting an overall trend toward a more aggressive clinical phenotype, although none of the analyzed clinicopathological variables reached statistical significance (Figure 3b–d). Overall, these findings suggest that elevated p38*α* expression may be associated with an unfavorable clinical outcome.

### 2.4. PDAC Cell Lines Display Heterogeneous but Overall Increased p-p38α Expression

To further explore the functional relevance of p38α activation, we examined the abundance of phosphorylated p38α levels across a panel of pancreatic cell lines representing different genetic backgrounds (Table 2). Notably, Western blot analysis revealed that almost all tumor cell lines, with the exception of AsPC-1, exhibited increased p-p38α levels compared with the non-tumorigenic pancreatic ductal epithelial cell line HPDE. Among the tumor cell lines, p38α activation was heterogeneous, with AsPC-1 displaying the lowest levels of phosphorylated p38α, whereas Capan-1, MiaPaCa-2, PANC-1, and BxPC-3 showed markedly higher activation (Figure 4a).

### 2.5. Pharmacological Inhibition of p38α Induces Apoptosis in PDAC Cell Lines

To determine whether p38α activation represents a therapeutically targetable vulnerability, high-p-p38α-expressing cell lines were treated with the selective p38α inhibitor ralimetinib. Ralimetinib is currently under clinical investigation for advanced malignancies and has been shown to suppress tumor growth and enhance sensitivity to chemotherapy [31]. The optimal concentration and exposure time for subsequent experiments were obtained by a dose–time response analysis in all four PDAC cell lines selected. As shown in supplementary Figure S1a, the IC50 was reached at a concentration of 10 μM after 48 h of treatment. Based on these results, the 10 μM concentration and 48h exposure time were selected for all subsequent experiments investigating the effects of pharmacological p38α inhibition on PDAC cell survival and apoptosis (Supplementary Figure S1a).

Treatment with ralimetinib resulted in a marked reduction in phosphorylation of MAPKAPK2 (p-MAPKAPK2), a well-established downstream effector of p38*α* signaling, confirming effective pathway inhibition (Figure 4b). Treatment with ralimetinib reduced pancreatic cancer cell viability (Figure 4c) and increased cell death (Figure 4d). To further investigate the mechanisms underlying the ralimetinib-induced reduction in pancreatic cancer cell viability, we assessed apoptosis by annexin V staining and flow cytometry analysis (Figure 4e, right panel). Importantly, ralimetinib treatment was associated with the induction of apoptosis (Figure 4e, left panel). Activation of the apoptotic pathway in pancreatic cancer cells treated with ralimetinib was further validated by immunoblotting experiments for cleaved PARP and other apoptotic cell markers (Figure 4f). These results support a pro-survival role of p38 signaling in PDAC cells. To further confirm the specific contribution of p38α to the observed phenotype, MAPK14 was genetically silenced using siRNA. Consistent with the effects observed following ralimetinib treatment, MAPK14 knockdown resulted in reduced p-MAPKAPK2 levels associated with the induction of apoptosis, thereby recapitulating the pharmacological inhibition phenotype (Supplementary Figure S1b). These findings provide complementary genetic evidence supporting a role for p38α in maintaining PDAC cell survival.

### 2.6. p38α Inhibition Impairs Tumorsphere Growth and Viability in 3D Models

Given the pre-clinical limitations of two-dimensional (2D) cultures, we next employed a three-dimensional (3D) tumorsphere model to better recapitulate tumor architecture and cell–cell interactions. Tumorspheres were generated from high-p-p38α PDAC cell lines and treated with or without ralimetinib.

Live/dead assays demonstrated a significant reduction in tumorsphere size following treatment, associated with a marked increase in red fluorescence intensity corresponding to BOBO-3 iodide uptake, a marker of apoptotic cell death (Figure 5a–c). These findings indicate that p38*α* inhibition reduces tumor cell viability and growth in a physiologically relevant 3D context.

### 2.7. Patient-Derived Organoids Confirm p38α as a Therapeutic Vulnerability in PDAC

To translate our findings into a more clinically relevant setting, patient-derived organoids were established from PDAC tumors (PDTOs) and matched adjacent normal pancreatic tissues (PDNOs).

Using Droplet digital PCR analysis, we firstly identified patients with increased p38α expression in tumor tissues compared with matched normal samples (Supplementary Figure S2).

Similarly to the findings in tumorspheres, treatment of selected PDTOs with ralimetinib induced both a significant size reduction and robust apoptotic cell death, as demonstrated by live/dead assays (Figure 6a–c). These results support a role for p38α signaling in maintaining the survival of PDTOs and highlight its inhibition as a promising therapeutic strategy.

Taken together, our multi-layered experimental approach, spanning transcriptomic profiling, tissue validation, in vitro models, and patient-derived organoids, provides convergent evidence that increased p38α signaling is associated with the invasive PDAC phenotype and functionally contributes to PDAC cell survival. The observation that the p38 MAPK pathway is less enriched in IPMN lesions than in invasive PDAC, together with the increased p-p38α levels observed in PDAC tissues, supports an association between p38α activation and malignant disease phenotype. Importantly, our functional data demonstrate that pharmacological inhibition of p38α reduces PDAC cell viability and induces apoptosis across two-dimensional and three-dimensional models, including patient-derived organoids. Overall, these findings identify p38α activation as a feature associated with PDAC and highlight its inhibition as a promising therapeutic strategy.

## 3. Discussion

PDAC is currently one of the most aggressive and lethal malignancies, characterized by profound resistance to currently available therapeutic options [35,36]. In this context, unraveling the molecular mechanisms underlying tumor progression is essential to identify novel therapeutic targets capable of effectively counteracting the biology of this disease.

Numerous studies have demonstrated that p38, a mitogen-activated protein kinase (MAPK) family member, plays a key role in various biological processes, including cellular responses to stress and inflammation, as well as the control of proliferation, differentiation, and survival [37,38]. Nevertheless, the precise contribution of p38 signaling to pancreatic cancer remains incompletely understood, largely due to the existence of four distinct isoforms (p38α, p38β, p38γ, and p38δ), which have been reported to exert divergent biological functions [27,37]. Among them, p38α appears to play a particularly complex and context-dependent role. During the early stages of tumorigenesis, p38α may act as a stress-response mediator that limits uncontrolled proliferation and preserves cellular homeostasis [39]. However, as malignant progression occurs, tumor cells can exploit p38α signaling to enhance survival, adapt to microenvironmental stress, sustain inflammatory responses, and develop resistance to chemotherapeutic agents [40].

Our study further supports an association between p38α signaling and pancreatic cancer progression. Indeed, our transcriptomic analyses revealed reduced p38 MAPK pathway enrichment in IPMN samples compared with PDAC tissues. In agreement with these findings, phosphorylation levels of p38α were significantly increased in PDAC specimens relative to precursor lesions, supporting an association between increased p38α activation and the invasive PDAC phenotype. Importantly, the IPMN and PDAC samples analyzed in this study were obtained from distinct patient groups and were not longitudinally collected from the same lesions. Therefore, these data do not establish p38α activation as a temporal or causal molecular event driving the transition from IPMN to invasive PDAC. Rather, they identify increased p38α signaling as a molecular feature associated with the invasive PDAC phenotype. Longitudinal analyses of lesions undergoing malignant transformation would be required to determine whether p38α activation precedes or accompanies this transition. Currently, surgical resection represents the only curative treatment option for eligible patients [6,41]. However, the extreme intratumoral heterogeneity of PDAC severely complicates the identification of effective pharmacological strategies, ultimately detrimentally affecting the overall clinical outcome [29]. In this scenario, the detection of p-p38α activation across different pancreatic cell lines, each characterized by a distinct genetic profile, suggests a functional dependence of PDAC on this pathway. This evidence opens a prospect of considerable clinical interest: p-p38α could represent a shared therapeutic target capable of overcoming the intrinsic limitations imposed by the high heterogeneity of this neoplasm. Indeed, the recent literature underscores that hyperactivity of the p38 signaling pathway serves as a major driver of chemoresistance in PDAC, acting both directly on tumor cells and indirectly by modulating the surrounding microenvironment [42].

The clinical development of numerous p38 inhibitors was terminated due to severe systemic toxicities or a dramatic lack of in vivo efficacy. These failures were often mispredicted by preclinical studies conducted exclusively on 2D monolayers, which fail to replicate the complex architecture and 3D biophysical stress typical of the PDAC microenvironment [28,43]. Importantly, we investigated the effects of pharmacological p38α inhibition using ralimetinib, a selective p38 inhibitor capable of attenuating stromal inflammation and restoring the sensitivity of tumor cells to chemotherapeutic drugs, which has been evaluated in clinical trials for other advanced-stage malignancies (e.g., NCT01663857, NCT01393990). These studies provided crucial data regarding its safety, manageability, and tolerability in patient-derived PDAC specimens, offering a solid rationale for investigating its therapeutic repositioning in PDAC. Indeed, in our study, we sought to overcome the 2D methodological limitation by validating the efficacy of ralimetinib in complex 3D biological systems. Our findings provide evidence for a functional role of p38α signaling in PDAC cell survival and demonstrate pharmacological sensitivity to p38α inhibition, accompanied by reduced MAPKAPK2 phosphorylation and increased apoptosis in both standard cultures and 3D spheroids. However, the downstream molecular mechanisms responsible for this dependency remain to be elucidated. Notably, the most significant findings emerged in PDAC patient-derived organoid models, which are valuable and clinically relevant ex vivo models that can recapitulate key features of patient tumors and may provide insights into patient-specific therapeutic responses. The fact that ralimetinib demonstrated marked antitumor activity by inducing apoptosis—as confirmed by PARP cleavage, Annexin V positivity, and BOBO-3 iodide incorporation—within these complex 3D architectures confers substantial translational relevance to our study.

## 4. Materials and Methods

### 4.1. Cell Lines

Human pancreatic cancer cell lines AsPC-1, Mia PaCa-2, BxPC3, Capan-1, and PANC-1 were obtained from the American Type Culture Collection (ATCC, Manassas, VA, USA). HPDE was purchased from Sigma (SCC442, Sigma-Aldrich, Milan, Italy). MiaPaCa-2 and PANC-1 cells were grown in DMEM high glucose (HG) without pyruvate (41965-039, Gibco, Bleiswijk, The Netherlands) with 10% FBS (A5256701, Gibco, Bleiswijk, The Netherlands) and 100 IU/mL penicillin–streptomycin (15140122, Gibco, Bleiswijk, The Netherlands). AsPC-1, BxPC3, and Capan-1 cells were cultured in RPMI high glucose without pyruvate (21875-034, Gibco, Bleiswijk, The Netherlands) with 10% FBS (A5256701, Gibco, Bleiswijk, The Netherlands) and 100 IU/mL penicillin–streptomycin (15140122, Gibco, Bleiswijk, The Netherlands). HPDE cells were cultured in Keratinocyte-SFM high L-Glutamine (10724-011, Gibco, Bleiswijk, The Netherlands) with 100 IU/mL penicillin–streptomycin (15140122, Gibco, Bleiswijk, The Netherlands). Cells were routinely propagated under standard conditions. All cell lines were tested to be mycoplasma-free (117048, Minerva Biolabs, Berlin, Germany) multiple times throughout the study. All cell cultures were performed in a 37 °C and 5% CO_2_ incubator.

### 4.2. Chemicals

Ralimetinib (LY2228820) was purchased from Selleckchem (S1494, Houston, TX, USA). Pancreatic cell lines, tumorspheres, and patient-derived organoids were treated for 48 h with ralimetinib 10 µM.

### 4.3. Immunohistochemistry

Human tissue specimens were fixed in 4% buffered formalin and embedded in paraffin. Formalin-fixed paraffin-embedded (FFPE) tissue blocks were obtained from 30 patients subdivided into two groups: IPMN group (*n* = 14) and PDAC group (*n* = 16); all patients underwent surgical resection. Sequential sections (4 μm) were cut, stained with hematoxylin and eosin (H&E), and used for morphological studies and immunohistochemical analysis. Sections were dewaxed and rehydrated in dH2O. Endogenous peroxidase activity was blocked by incubation in 3% hydrogen peroxide for 10 min. Then, sections were mounted on Apex Bond IHC Slides (3800040, Leica Biosystems, Deer Park, IL, USA) and used for immunohistochemical analysis. Immunohistochemical staining procedures were carried out on a DAKO OMNIS 165 DUO automated immunostainer (Agilent Technologies, Zürich, Switzerland), from deparaffinization to counterstaining with hematoxylin, using EnVision FLEX High pH Kit (GV823, Dako Omnis, Agilent Technologies, Zürich, Switzerland). Then, sections were incubated overnight with Monoclonal Antibody p-p38α (Thr180/Tyr182) (MA5-15177, Thermo Fisher Scientific, Waltham, MA USA) as primary antibody (1:200). Antigen retrieval was performed using the Target Retrieval Solution, High pH (50× Tris/EDTA buffer, pH 9) (K8004, Dako Omnis, Agilent Technologies, Santa Clara, CA USA). Negative controls were used in each experiment. Expression evaluation was performed using the H-score method (low, moderate, and high expression). Images were acquired using the NanoZoomer S20 Digital Slide Scanner (Hamamatsu Photonics, Arese, Italy).

Scoring of staining: scoring of 30 IPMN and PDAC tissue sections were assessed by using the semi-quantitative pathology histoscore (H-score), defined as a method combining both percentages of positive expression cells in the tissue slice and immunostaining intensities (hereinafter referred to as IHC-score). The IHC score was based on the staining intensity level of p-p38α from 0 to 1+ (weak), 2+ (intermediate), or 3+ (strong). In brief, the H-score was calculated according to the formula: (0 × percentage of immunonegative cells) + (1 × percentage of weakly stained cells) + (2 × percentage of intermediately stained cells) + (3 × percentage of strongly stained cells). Thus, the H-score ranged from 0 (a tissue sample that is completely negative) to a maximum of 300 (a tissue sample in which all the cells show a 3+ staining), separating more distinctly the samples with predominantly high staining intensity from samples with a predominantly low staining intensity [44]. A sample was considered positive with an H-score ≥ 5 [45]. All samples of this study were assessed by two pathologists working independently. In case of discrepancies in the assessments, the sections were discussed to reach a final agreement. H-score method (low, moderate, and high expression).

### 4.4. Live and Dead Assay

For PDTO and spheroids experiments, three independent biological experiments were performed, with individual organoids and spheroids analysed per condition in each experiment. The area and red fluorescence of each organoid and spheroid were measured, and the means were calculated for each condition within each independent experiment. The 3 independent experiments were considered biological replicates. Since control and ralimetinib-treated samples were analysed in parallel within each independent experiment, the mean values were paired by experiment, and comparisons were performed using a two-tailed paired Student’s *t*-test. *p* < 0.05 was considered statistically significant. Tumorspheres from pancreatic cancer cell lines and PDTOs were stained using the LIVE/DEAD^®^ Cell Imaging Kit (R37601, Thermo Fisher Scientific, Bleiswijk, The Netherlands) according to the manufacturer’s instructions. Specifically, live cells were stained green (Calcein AM), and dead cells were stained red (BOBO-3 Iodide). Digital image acquisition was carried out with a Zeiss Axio Observer fluorescence microscope (Carl Zeiss, Jena, Germany) using a 5× magnification objective. Area and intensity of red fluorescence were analysed using ZEN blue software 3.3 version (Carl Zeiss, Jena, Germany).

### 4.5. Immunoblotting

Whole-cell extracts were obtained from pancreatic cell lines. Cells were collected and homogenized in lysis buffer (50 mM Tris-HCl pH 7.4, 5 mM EDTA, 250 mM NaCl, and 1% Triton X-100) supplemented with protease and phosphatase inhibitors (78443, Thermo Fisher Scientific, Bleiswijk, The Netherlands). In total, 20 μg of protein extracts from each sample was denatured in 4× Laemmli sample buffer (1610747, Bio-Rad Laboratories, Hercules, CA, USA) and subjected to 4–15% and 7.5% precast TGX Stain-Free polyacrylamide gel (4568024, Bio-Rad Laboratories, Hercules, CA, USA) electrophoresis. Proteins were electrotransferred onto a nitrocellulose membrane and blocked with 1× PBS with 1% casein (1610783, Bio-Rad Laboratories, Hercules, CA, USA) for 40 min at room temperature for immunoblot analysis. Primary antibodies used: p-p38α (MA5-15177, Thermo Fisher Scientific, Waltham, MA USA), p38α (9218, Cell Signaling Technology, Danvers, MA, USA), p-MAPKAPK-2 (3007, Cell Signaling Technology, Danvers, MA, USA), Cleaved PARP (5625, Cell Signaling Technology, Danvers, MA, USA), Cleaved Caspase-8 (9496, Cell Signaling Technology, Danvers, MA, USA), Cleaved Caspase-3 (9661, Cell Signaling Technology, Danvers, MA, USA), Cytochrome c (4280, Cell Signaling Technology, Danvers, MA, USA), VINCULIN (13901, Cell Signaling Technology, Danvers, MA, USA). Rabbit IgG HRP (1706515, Bio-Rad Laboratories, Hercules, CA, USA) was used as a secondary antibody and revealed using the Clarity Max Western ECL Substrate (1705062, Bio-Rad Laboratories, Hercules, CA, USA).

### 4.6. Tumorsphere Formation Efficiency Assay

Cells (500/well) were seeded into ultra-low attachment 24-well cluster plates (3473, Costar Corning, Kennebunk, ME, USA) and cultured in Advanced DMEM/F-12 (12634010, Gibco, Bleiswijk, The Netherlands) supplemented with 1% L-glutamine (25030081, Gibco, Bleiswijk, The Netherlands), 100 IU/mL penicillin–streptomycin (15140122, Gibco), 0.6% glucose solution (G8769, Sigma-Aldrich, Milan, Italy), B-27 (12587010, Gibco, Bleiswijk, The Netherlands), N-2 (17502048, Gibco, Bleiswijk, The Netherlands), 10 ng/mL HbFGF (F0291, Sigma-Aldrich, Milan, Italy), 20 ng/mL EGF (E9644, Sigma-Aldrich, Milan, Italy). After two days in culture, spheres were photographed, counted, and analyzed daily.

### 4.7. Patient Samples

A total of 30 patients who underwent surgical resection at our institution were included in the study, comprising 14 patients with diagnosis of IPMN and 16 patients with PDAC. Patients were consecutively identified according to the availability of adequate tissue specimens for molecular and histopathological analyses. The diagnosis was established by histopathological examination. Cases were excluded when insufficient or inadequate tissue material was available for the planned analyses. The study was conducted in accordance with the Declaration of Helsinki and was approved by the Ethics Committee of the Istituto Tumori “Giovanni Paolo II” (Prot. n. 270/C.E.; approval date: 18 May 2023). Written informed consent was obtained from all participants.

### 4.8. RNA Extraction

Total RNA was extracted from a FFPE section of 10 μm thickness. RNA purification was carried out using the RNeasy FFPE Kit (73504, Qiagen, Hilden, Germany) according to the manufacturer’s protocol, including the treatment of sections with Deparaffinization Solution (19093, Qiagen, Hilden, Germany). The RNA was finally eluted in 30 μL of ribonuclease-free water. The RNA concentration was determined with the NanoDrop ND-2000 Spectrophotometer (Nanodrop Technologies, Wilmington, DE, USA).

### 4.9. RNA Sequencing and Gene Set Enrichment Analysis

RNA sequencing was performed on 30 human pancreatic tissue samples using the Illumina NovaSeq 6000 platform with paired-end 2 × 101 bp sequencing. Raw sequencing reads were subjected to quality control using FastQC (version 0.12.0), followed by adapter trimming and removal of low-quality reads and reads shorter than 25 bp using Cutadapt (v4.4). Processed reads were aligned to the human reference genome (HG38, GENCODE release 44) using STAR (v2.7.10b) with default parameters for paired-end sequencing. Deregulated expression analysis was performed by Genomix4Life (Baronissi (SA), Italy) in R (version 4.5.0) using the DESeq2 (version 1.48.2) Bioconductor package. Gene Set Enrichment Analysis (GSEA) was performed in R (version 4.5.0) using the clusterProfiler package. Genes were ranked according to the Wald statistic obtained from the DESeq2 expression analysis. GSEA was conducted using Gene Ontology Biological Process (GO-BP) gene sets from the org.Hs.eg.db annotation database, with gene symbols as identifiers (keyType = “SYMBOL”). Gene sets containing between 10 and 500 genes were included in the analysis, and statistical significance was evaluated using nominal *p* values and false discovery rate (FDR)-adjusted *p* values to account for multiple testing. Enrichment results were visualized using ridge plot generated with the clusterProfiler (version 4.15.2), ggplot2 (version 4.0.3), and ggridges (version 0.5.7) packages. Specifically, the GO:0038066 “p38MAPK cascade” gene set was used to define p38 MAPK pathway enrichment. The RNA-seq dataset generated in this study is publicly available in the Gene Expression Omnibus (GEO) repository under accession number [GSE347363].

### 4.10. TCGA Survival Analysis

Clinical and gene expression data from patients with pancreatic adenocarcinoma (PAAD) were obtained from The Cancer Genome Atlas (TCGA). MAPK14 mRNA expression values were extracted from the RNA sequencing dataset and patients were stratified into high and low expression groups according to the median MAPK14 expression level. Overall survival was analyzed using the Kaplan–Meier method implemented in the R packages survival and survminer. Survival differences between groups were assessed using the log-rank test, and *p* values < 0.05 were considered statistically significant.

### 4.11. Clinicopathological Association Analysis

Associations between p38α expression and clinicopathological characteristics were evaluated using TCGA-PAAD clinical annotations. Patients were stratified into high and low p38α expression groups according to the median expression value. The distribution of clinicopathological variables across expression groups was visualized using stacked bar plots showing the percentage of patients in each category.

### 4.12. Droplet Digital PCR (ddPCR) Assay

ddPCR was performed using 10 ng of RNA extracted from tissue samples. RNA was extracted as previously described and retro-transcribed to cDNA using the SuperScript™ IV VILO™ Master Mix (11756050, Thermo Fisher Scientific, Baltics UAB, Vilnius, Lithuania) according to the manufacturer’s instructions. Reactions were prepared using the ddPCR EvaGreen Supermix (1864033, Bio-Rad Laboratories, Hercules, CA, USA) and specific primers according to the manufacturer’s instructions. Droplets were processed as described above and amplified under the following cycling protocol: a denaturation step at 95 °C for 5 min, followed by 40 cycles at 95 °C for 30 s and 60 °C for 1 min, a postcycling step for signal stabilization at 4 °C for 5 min and 95 °C for 5 min, and infinite hold at 4 °C. The plate was then transferred into a QX200 Reader (Bio-Rad Laboratories, Hercules, CA, USA). Data were analyzed using Bio-Rad QX Manager 1.2 Standard Edition. ddPCR primer sequences are available upon request.

### 4.13. Patient-Derived Organoids

Patient-derived organoids were generated from patients’ biopsies according to PancreaCult™ Organoid Media (Human) product information sheet (100-0820, StemCell Technologies, Vancouver, BC, Canada). Briefly, tissues were dissociated using the gentle cell dissociation reagent (100-0485, StemCell Technologies, Vancouver, BC, Canada); 4 × 10^3^ dissociated crypts were mixed into a DMEM/F-12 (31330038, Gibco, Bleiswijk, The Netherlands) and Matrigel Matrix (356234, Costar Corning, Kennebunk, ME, USA) solution at a 1:1 volume ratio in 24-well TC-treated plates (3526, Costar Corning, Kennebunk, ME, USA) at 37 °C until Matrigel solidification. Then, 750 μL of PancreaCult™ Organoid Media (Human) (100-0820, StemCell Technologies, Vancouver, BC, Canada) was added on top of the Matrigel dome. Patient-derived organoids were passaged every 7–10 days.

### 4.14. Cell Viability

Cell viability was assessed using the CellTiter-Glo Luminescent Cell Viability Assay Kit (G7570, Promega, Madison, WI, USA), according to the manufacturer’s instructions. Briefly, a total of 2 × 10^3^ dissociated cells were plated in 96-well plates. After 24 h, cells were treated with or without ralimetinib 2.5, 5, 10 and 20 μM for 24, 48 and 72 h. Then, cells were processed according to the manufacturer’s instructions. The luminescent signal was read using the SPECTROstar Omega microplate reader (BMG Labtech, Offenburg, Germany).

### 4.15. Cell Death

Cell death was assessed by counting. Briefly, a total of 2 × 10^3^ dissociated cells were plated in 96-well plates (3474, Costar Corning, Kennebunk, ME, USA). After 24 h, cells were treated or not with ralimetinib (10 μM, 48 h). Then, supernatants (containing dead/floating cells) were collected. Cell pellets were resuspended in 1× PBS, and 10 μL was mixed with an equal volume of 0.01% Trypan blue solution (T8154, Sigma-Aldrich, Milan, Italy). Viable cells (unstained, trypan blue-negative cells) and dead cells (stained, trypan blue-positive cells) were counted with a phase-contrast microscope, and the percentages of dead cells were calculated.

### 4.16. Annexin V Staining

For this assay, 1 × 10^6^ cells were cultured in 6-well plates (3471, Costar Corning, Kennebunk, ME, USA) at 37 °C, 5% CO_2_. After 24 h, cells were treated with or without ralimetinib. Next, 5 × 10^4^ cells/plate were collected and resuspended in 1× PBS-1% FBS, and the Muse Annexin V and Dead Cell reagent (MCH100105, Luminex, Austin, TX, USA) was added to each tube. Cells were then incubated at room temperature for 20 min in the dark. Flow cytometry was performed using the Guava Muse Cell Analyzer (Merck Millipore). Cells were considered to be apoptotic if they were Annexin V +/PI− (early apoptotic) and Annexin V +/PI+ (late apoptotic). Each analysis was performed by evaluating at least 2000 events, using the assay-specific software module included in the Guava Muse Cell Analyzer instrument (Merck Millipore, Darmstadt, Germany).

### 4.17. siRNA Transfection

Mia PaCa-2, BxPC3, Capan-1, and PANC-1 cells were transfected with 10 nM validated siRNA directed against p38α (s3585, Ambion, Carlsbad, CA, USA) using the HiPerfect reagent (301704, QIAGEN, Hilden, Germany) according to the manufacturer’s instructions. Silencer™ Select Negative Control (4390843, Life Technologies, Carlsbad, CA, USA) was used as a control.

### 4.18. Quantification and Statistical Analysis

The statistical significance of the results was analysed using Student’s *t*-test, and * *p* < 0.05 was considered statistically significant.

## 5. Conclusions

In conclusion, our findings provide preclinical evidence supporting p38α as a potentially actionable target in PDAC and provide a rationale for further investigation of p38α inhibition. Ralimetinib-mediated inhibition of p38α reduced pathway activity and promoted apoptotic cell death across multiple experimental models, including patient-derived tumor organoids. However, although ralimetinib is described as a selective p38α inhibitor, we acknowledge that, at the concentration used in our study (10 μM), potential off-target effects cannot be fully excluded. Therefore, the pharmacological effects observed should be interpreted as evidence of sensitivity to ralimetinib and as consistent with a potential contribution of p38α signaling, while acknowledging that additional studies are needed to further define the specificity of this effect. Several limitations of the present study should be acknowledged. Although the use of multiple complementary models strengthens our findings, these models cannot fully recapitulate the complexity and heterogeneity of human PDAC, and the response observed in patient-derived organoids cannot be considered predictive of clinical benefit. Therefore, further preclinical studies, including in vivo validation, followed by well-designed clinical studies, will be necessary to determine the therapeutic efficacy, safety, and optimal clinical application of p38α inhibition in PDAC. Given the increasing recognition that effective cancer treatment relies on rational multi-target therapeutic strategies rather than single-agent approaches, our findings suggest that p38α inhibitors may represent valuable components of combination regimens designed to improve therapeutic efficacy in PDAC. These possibilities, however, require experimental validation before any conclusions regarding their clinical utility can be drawn.

## Figures and Tables

**Figure 1:**
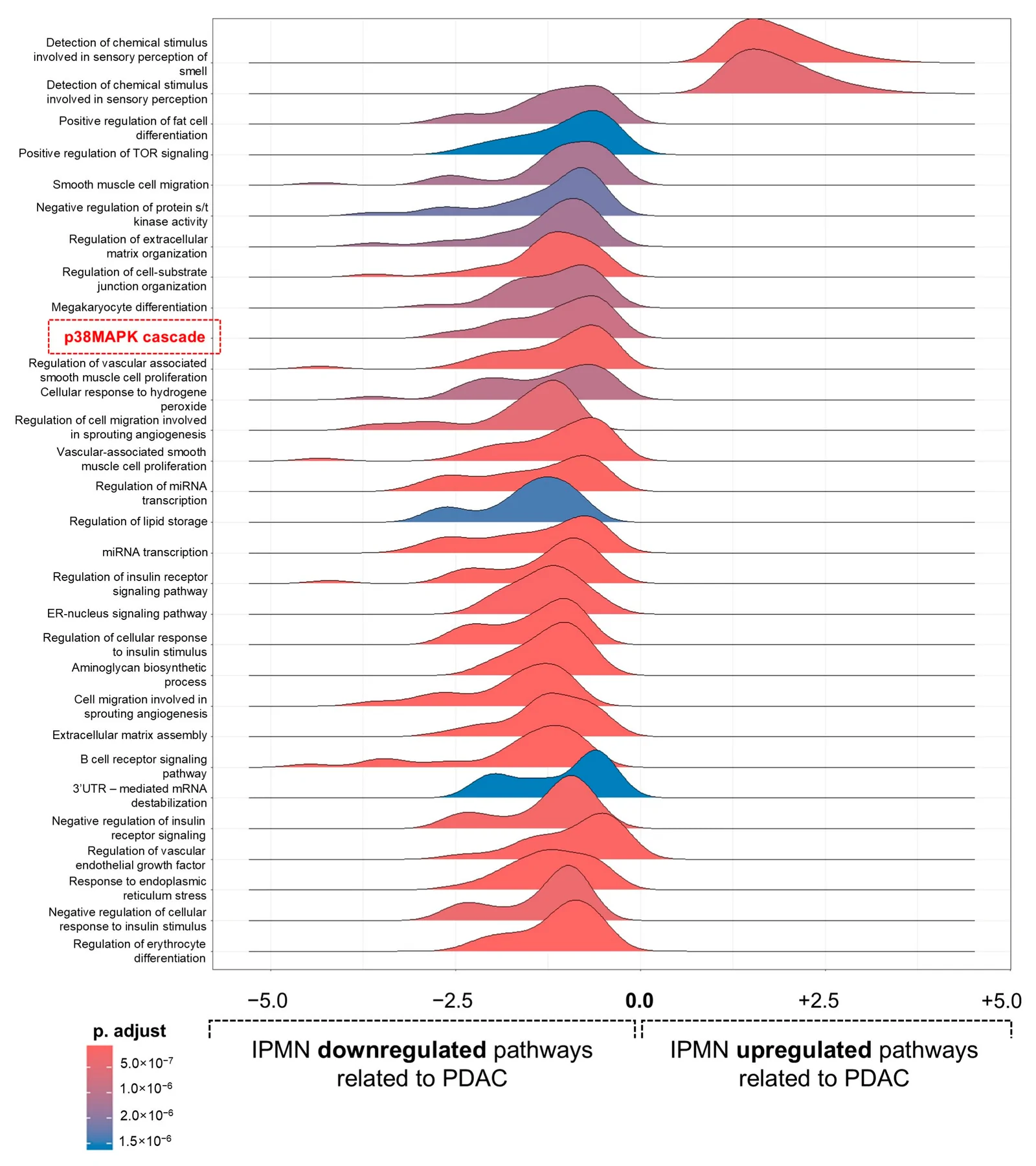
Gene set enrichment analysis between IPMN and PDAC. Gene Ontology (GO) Biological Process (BP) gene set enrichment analysis of genes ranked according to the Wald statistic obtained from the differential expression analysis of IPMN versus PDAC samples. The ridge plot displays significantly enriched GO-BP terms. Ridge density represents the distribution of gene-level ranking statistics for genes belonging to each GO term. The pathway highlighted in red represents the pathway of interest discussed in the results.

**Figure 2:**
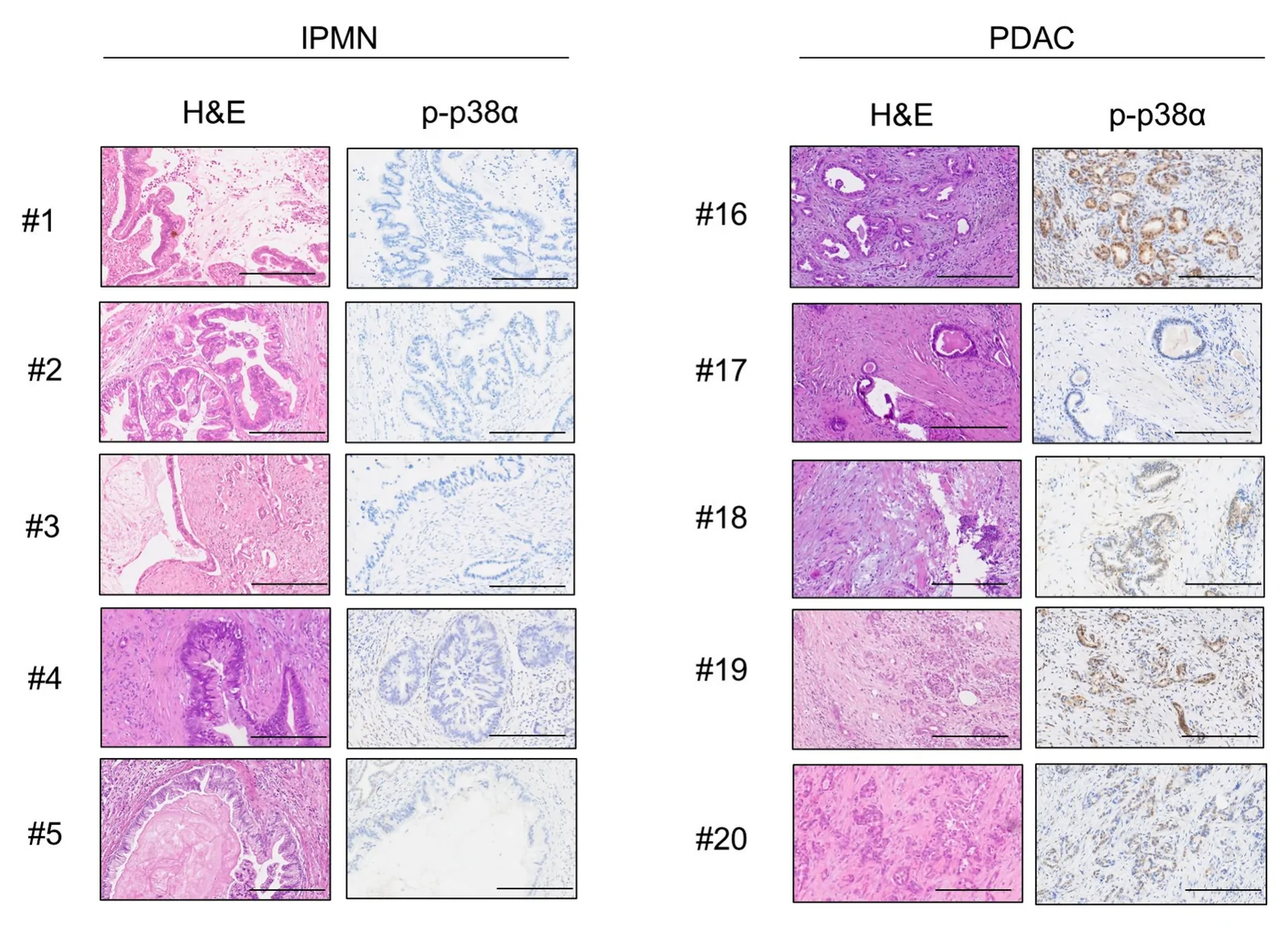
Phosphorylated p38α level is increased in PDAC compared with IPMN. Representative hematoxylin and eosin (H&E) staining and immunohistochemical (IHC) analysis of phosphorylated p38α (p-p38α) in intraductal papillary mucinous neoplasms (IPMNs) and pancreatic ductal adenocarcinoma (PDAC) specimens. Representative H&E and corresponding p-p38α immunostaining of IPMN samples (**left panels**) showing weak or absent p38α activation and of PDAC tissues (**right panels**) demonstrating increased p-p38α expression. Scale bars = 250 μm.

**Figure 3:**
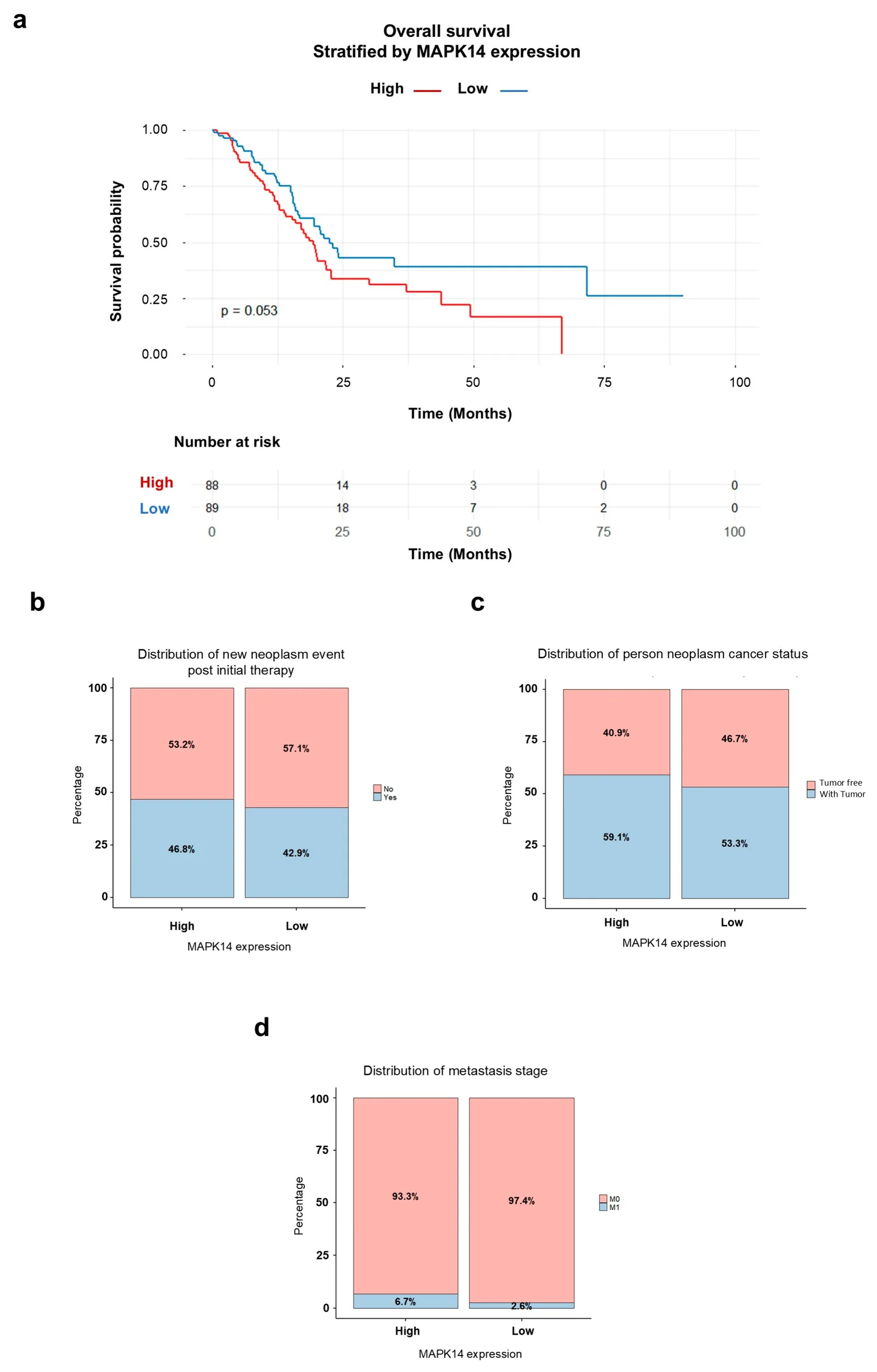
High MAPK14 expression is associated with a trend toward worse disease outcome in patients with pancreatic ductal adenocarcinoma. (**a**) Kaplan–Meier overall survival analysis of patients with pancreatic ductal adenocarcinoma (PDAC) from the TCGA-PAAD cohort stratified into p38*α*-high and p38*α*-low expression groups according to the median MAPK14 mRNA expression level. The number of patients at risk is reported below the survival curves. (**b**) Distribution of new neoplastic events following initial therapy according to MAPK14 expression. The proportion of patients developing a new neoplastic event (“Yes”) or remaining event-free (“No”) is shown for tumors with high and low MAPK14 expression. (**c**) Distribution of person neoplasm cancer status according to MAPK14 expression. The percentage of patients classified as tumor-free or with residual/persistent tumor at the time of follow-up is reported for the high and low MAPK14 expression groups. (**d**) Distribution of metastatic stage according to MAPK14 expression. The relative proportions of patients with non-metastatic (M0) and metastatic (M1) disease are shown in tumors with high and low MAPK14 expression. Stacked bar plots represent the percentage of patients within each expression group.

**Figure 4:**
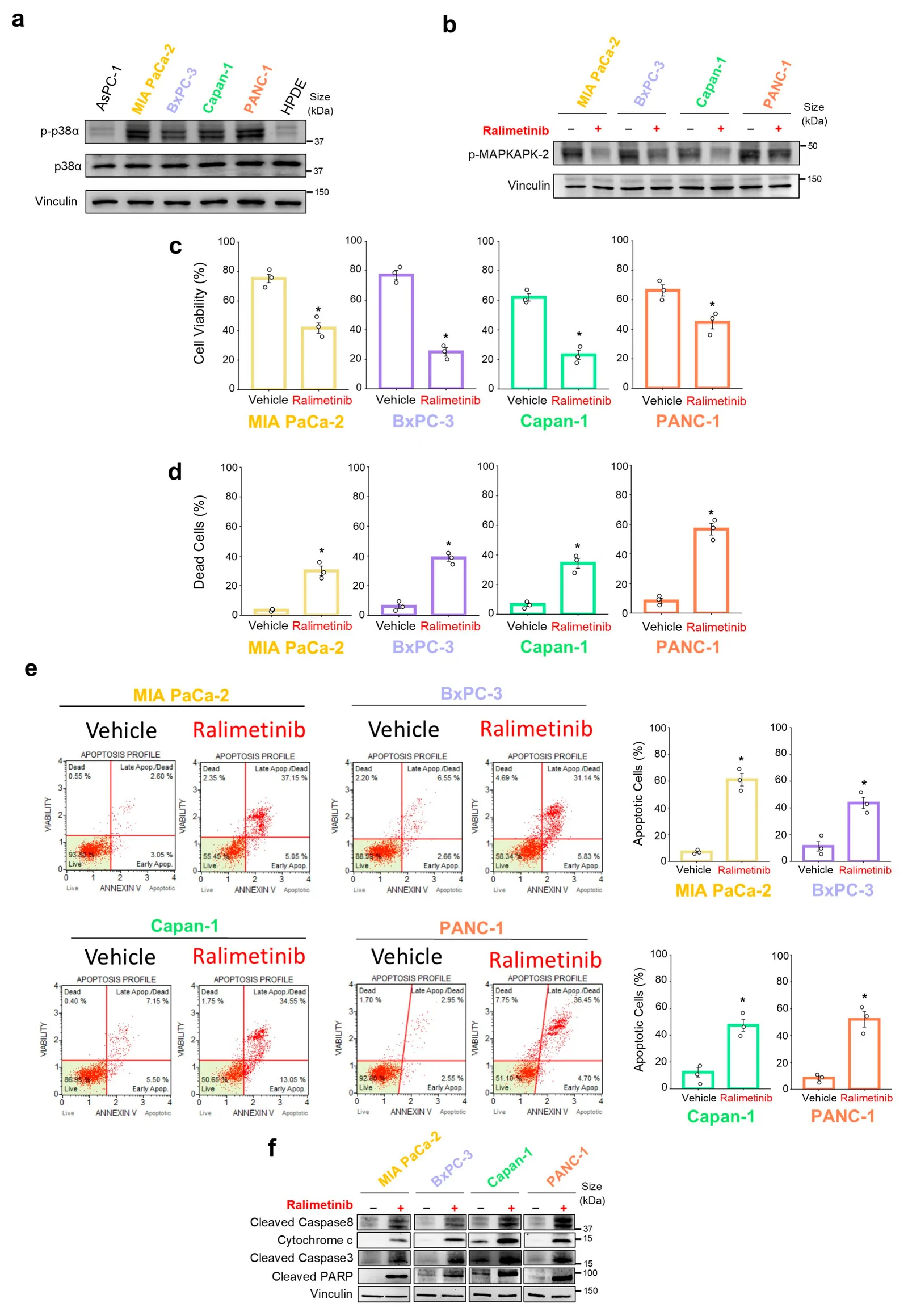
Basal p38α activation differs among pancreatic cancer cell lines. (**a**) Representative Western blot analysis of phosphorylated p38α (p-p38α) and total p38α expression in the normal pancreatic epithelial cell line (HPDE) and the indicated pancreatic ductal adenocarcinoma (PDAC) cell lines. VINCULIN was used as a loading control. (**b**) PDAC cell lines were treated with ralimetinib (10 μM, 48 h). VINCULIN was used as a loading control. (**c**) Cell viability analysis of MIA PaCa-2, BxPC-3, Capan-1, and PANC-1 cells treated with vehicle or ralimetinib (10 μM, 48 h). (**d**) Quantification of dead cells following treatment with vehicle or ralimetinib (10 μM) for 48 h in the indicated PDAC cell lines. Data are presented as the percentage of dead cells. (**e**) Representative Annexin V/7-AAD flow cytometric dot plots showing apoptotic cell death in MIA PaCa-2, BxPC-3, Capan-1, and PANC-1 cells after 48 h of treatment with vehicle or ralimetinib (10 μM). Histograms report the percentage of Annexin V-positive apoptotic cells. (**f**) Representative Western blot analysis of PDAC cell lines following treatment with vehicle or ralimetinib (10 μM, 48 h). VINCULIN was used as a loading control. * *p* < 0.05 ralimetinib-treated vs. vehicle. Data are expressed as means ± SEM of 3 independent experiments. The red lines indicate the gating boundaries used to discriminate the viable cell population, highlighted by the green box, from the apoptotic populations detected within the remaining gates. Moreover, distinct colors are used throughout the text to clearly distinguish the four different cell lines.

**Figure 5:**
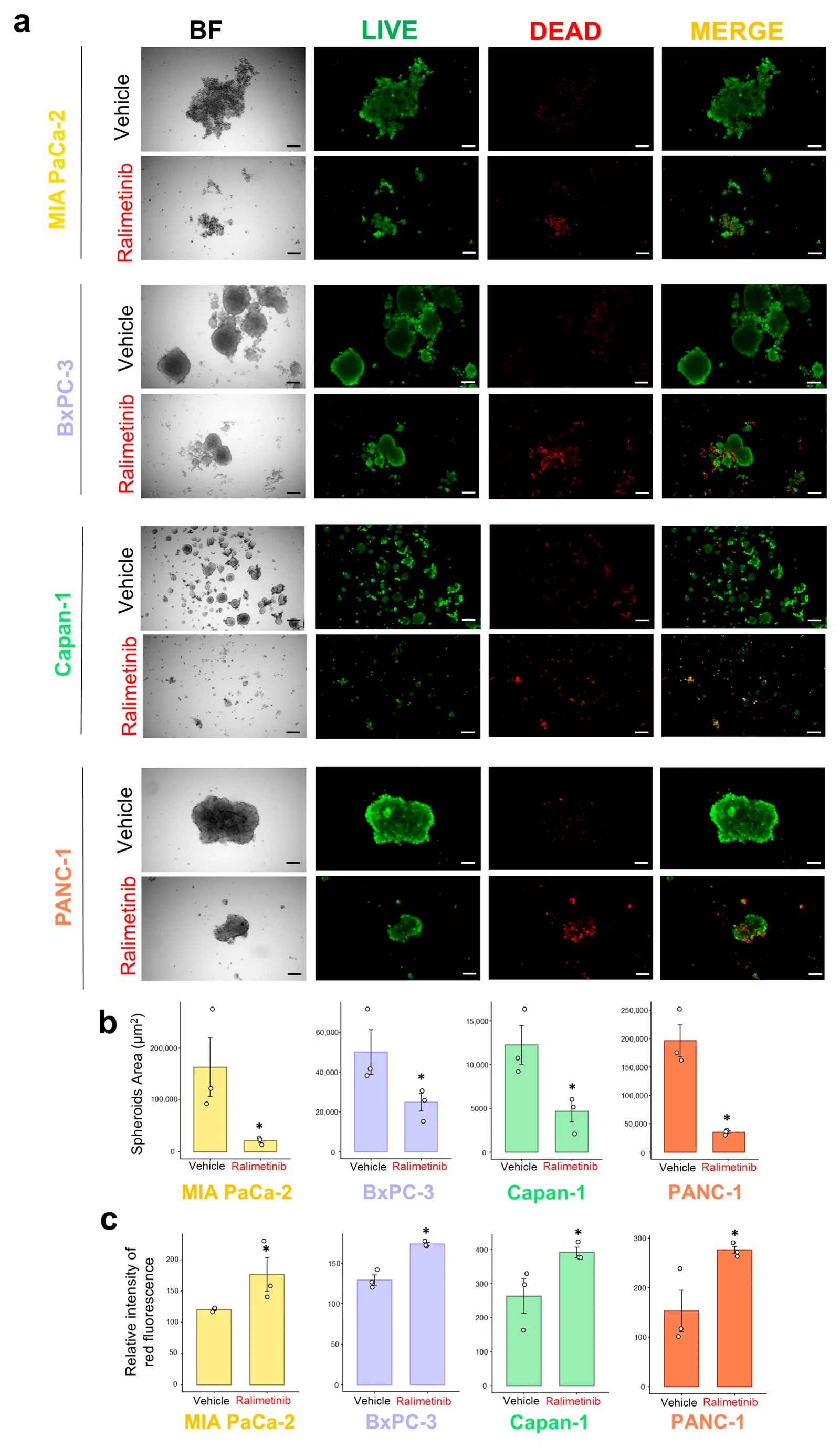
Ralimetinib reduces tumorsphere formation and increases cell death in pancreatic cancer 3D models. (**a**–**c**) Tumorsphere formation assay of pancreatic cancer three-dimensional (3D) models treated with or without the selective p38α inhibitor ralimetinib (10 μM, 48 h). Representative tumorsphere bright-field imaging and live and dead staining (green: live; red: dead cells) (**a**) (scale bar: 200 µm), area (**b**), and relative intensity of red fluorescence (**c**). Data are presented as mean ± SEM from 3 independent biological experiments, with individual spheroids analyzed per condition in each experiment. For each independent experiment, the mean spheroid area and red fluorescence were calculated. Statistical significance was assessed using a two-tailed paired Student’s *t*-test (* *p* < 0.05). Comparisons were performed between vehicle and ralimetinib-treated conditions within each cell line.

**Figure 6:**
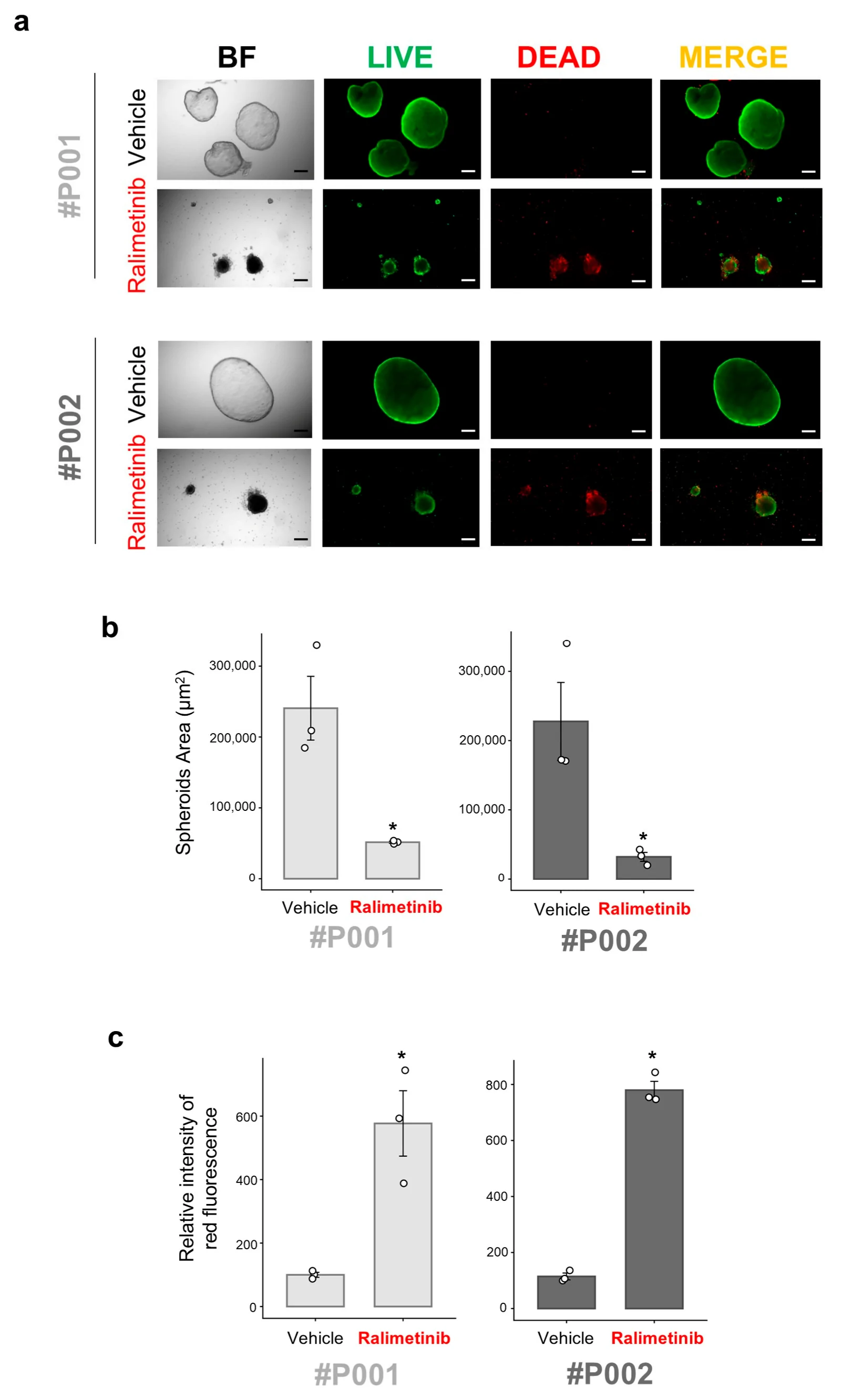
Pharmacological inhibition of p38α by ralimetinib impairs the viability of patient-derived pancreatic cancer organoids (PDTOs). (**a**–**c**) PDTOs treated with or without the selective p38α inhibitor ralimetinib (10 μM, 48 h). Representative PDTOs bright-field imaging and live/dead staining (green: live; red: dead cells) (**a**) (scale bar: 200 µm), quantification of organoid area (**b**), and relative intensity of red fluorescence (**c**). Data are presented as mean ± SEM from 3 independent biological experiments, with individual organoids analyzed per condition in each experiment. For each independent experiment, the mean organoid area and red fluorescence were calculated. Statistical significance was assessed using a two-tailed paired Student’s *t*-test (* *p* < 0.05). Comparisons were performed between vehicle and ralimetinib-treated conditions within each PDTO.

**Table 1:** Clinical and pathological characteristics of patients included in the study.

Patient’s Code	IPMN/PDAC	Gender	Age at Diagnosis	IPMN Dysplasia Grade	IPMN Subtype	Immuno Phenotype	Molecular Profile	MSI Status	H-Score p-p38α	Tumor Location	TNM	Grade	Invasion of Superior Mesenteric Area	Duodenal Invasion	Lymphatic Infiltration and Vascular Permeation	Perineural Infiltration	Death
#1	IPMN	F	72	High	Bilio pancreatic	CK7+			Neg	Head			No	Yes	Yes	Yes	Yes
#2	IPMN	M	61	High	Bilio pancreatic	CK7+			Neg	Head			Yes	Yes	Yes	Yes	Yes
#3	IPMN	M	68	High	Bilio pancreatic	CK7+			Neg	Tail			Yes	Yes	Yes	Yes	Yes
#4	IPMN	M	75	High	Intestinal	CDX2+			1+	Head			No	No	Yes	Yes	No
#5	IPMN	M	68	High	Bilio pancreatic	CK7+			Neg	Tail			No	No	Yes	Yes	No
#6	IPMN	F	73	Low moderate	Intestinal	CDX2+			Neg	Head			No	Yes	Yes	Yes	Yes
#7	IPMN	F	76	High	Gastric foveolar	MUC5AC+			Neg	Head			Yes	No	Yes	Yes	Yes
#8	IPMN	M	64	High	Intestinal	CDX2+			1+	Head			Yes	Yes	Yes	Yes	Yes
#9	IPMN	M	82	High	Gastric intestinal	MUC5AC+			Neg	Head			No	Yes	Yes	Yes	Yes
#10	IPMN	M	54	Moderate high	Pancreatic gastric	CK7+			Neg	Head			No	No	No	No	No
#11	IPMN	F	86	Moderate high	Gastric foveolar	MUC5AC+			Neg	Head			No	Yes	Yes	Yes	No
#12	IPMN	M	73	High	Intestinal mucinous	CDX2+			Neg	Head			No	No	Yes	Yes	Yes
#13	IPMN	F	78	High	Gastric foveolar	MUC5AC+			1+	Head			Yes	No	Yes	Yes	Yes
#14	IPMN	F	78	High	Intestinal	CDX2+			Neg	Head			Yes	Yes	Yes	Yes	No
#15	IPMN	F	65	High	Intestinal	CDX2+			Neg	Tail			Yes	Yes	Yes	Yes	No
#16	PDAC	M	62				G12D	MSS	3+	Head	T2N1M0	G2	Yes	No	Yes	Yes	No
#17	PDAC	M	71				G12V	MSS	2+	Head	T1cN0M0	G2	Yes	No	Yes	Yes	Yes
#18	PDAC	M	84				G12D	MSS	2+	Head	T2N1M0	G2	Yes	No	Yes	Yes	Yes
#19	PDAC	F	68				G12D	MSS	3+	Tail	T1cN0M0	G3	No	No	Yes	Yes	No
#20	PDAC	F	79				G12V	MSS	3+	Head	T2N1M0	G2	No	Yes	Yes	Yes	Yes
#21	PDAC	M	77				G12D	MSS	2+	Head	T1cN1M0	G3	Yes	No	Yes	Yes	Yes
#22	PDAC	M	70				G12D	MSS	2+	Head	T1N0M0	G3	No	Yes	Yes	Yes	No
#23	PDAC	M	50				G12V	MSS	2+	Head	T1cN0M0	G3	No	No	Yes	Yes	Yes
#24	PDAC	F	76				G12V	MSS	3+	Head	T2N2M0	G2	Yes	Yes	Yes	Yes	No
#25	PDAC	F	56				G12D	MSS	2+	Head	T1cN1M0	G3	Yes	Yes	Yes	Yes	Yes
#26	PDAC	F	77				G12R	MSS	3+	Head	T2N0M0	G3	Yes	No	Yes	Yes	Yes
#27	PDAC	M	81				G12V	MSS	3+	Head	T4N2M0	G3	Yes	Yes	Yes	Yes	Yes
#28	PDAC	M	63				G12D	MSS	3+	Head	T2N2M0	G3	No	Yes	Yes	Yes	Yes
#29	PDAC	F	75				G12D	MSS	3+	Head	T2N0M0	G2	No	Yes	Yes	Yes	Yes

**Table 2:** Origin and genetic profile of pancreatic cancer cell lines used in this study.

Cell Line	Histology	Tumor Source	Mutant Gene	Protein Sequence
AsPC-1	Adenocarcinoma	Metastasis, Ascites	KRAS	p.G12D
			TP53	p.C135AfsTer35
			SMAD4	p.R100T
			CDKN2A	p.L78HfsTer41
MIA PaCa-2	Adenocarcinoma	Primary	KRAS	p.G12C
			TP53	p.R248W
BxPC3	Adenocarcinoma	Primary	TP53	p.Y220C
Capan-1	Adenocarcinoma	Metastasis, liver	KRAS	p.G12V
			TP53	p.A159V
			SMAD4	p.S343Ter
PANC-1	Adenocarcinoma	Primary	KRAS	p.G12D
			TP53	p.R273H
HPDE	Ductal epithelium	N.A.	N.A.	N.A.

N.A. = Not applicable.

## Data Availability

The RNA-seq dataset generated in this study is publicly available in the Gene Expression Omnibus (GEO) repository under accession number (GSE347363).

## Supplementary Materials

The following supporting information can be downloaded at: https://www.mdpi.com/article/10.3390/ijms27198550/s1.

## Abbreviations

The following abbreviations are used in this manuscript:

CT	Computed tomography
IPMNs	Intraductal papillary mucinous neoplasms
MAPK	Mitogen-activated protein kinase
MRI	Magnetic resonance imaging
PDAC	Pancreatic ductal adenocarcinoma
PDNOs	Patient-derived normal organoids
PDTOs	Patient-derived tumor organoids
2D	Two-dimensional
3D	Three-dimensional
