# Supplementary material for: p38α Signaling Is Associated with Pancreatic Ductal Adenocarcinoma Progression and Represents a Potential Therapeutic Vulnerability
Source: Int J Mol Sci. 2026 Sep 24;27(19):8550. doi: 10.3390/ijms27198550 (PMC13665038; doi:10.3390/ijms27198550)

Supplementary Figure S1

a

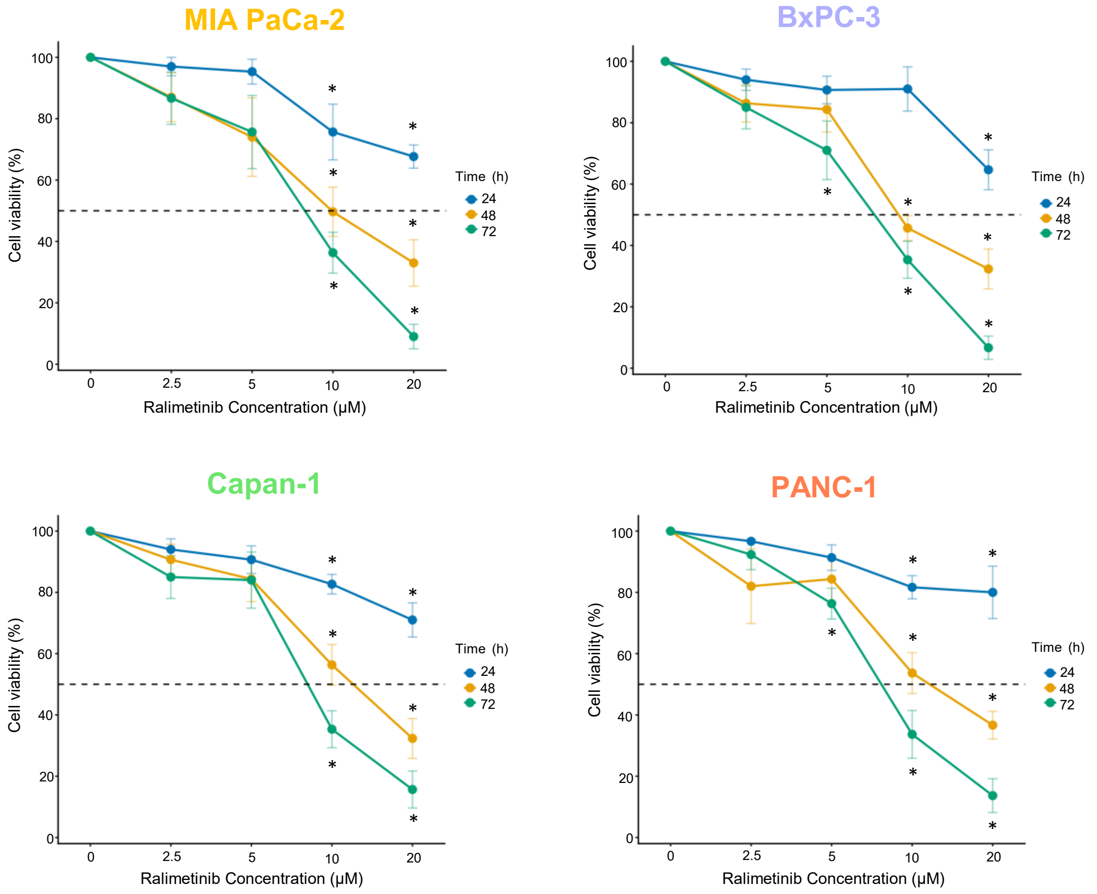

b

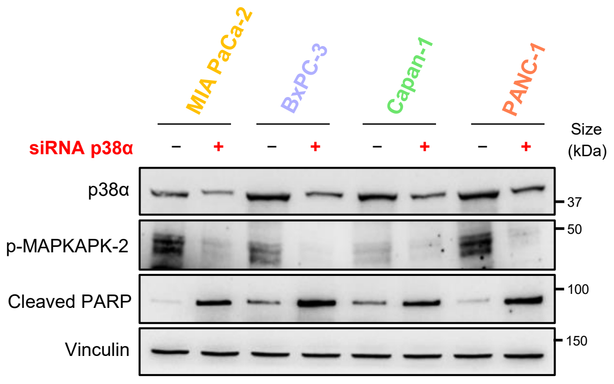

Supplementary Figure S2

a

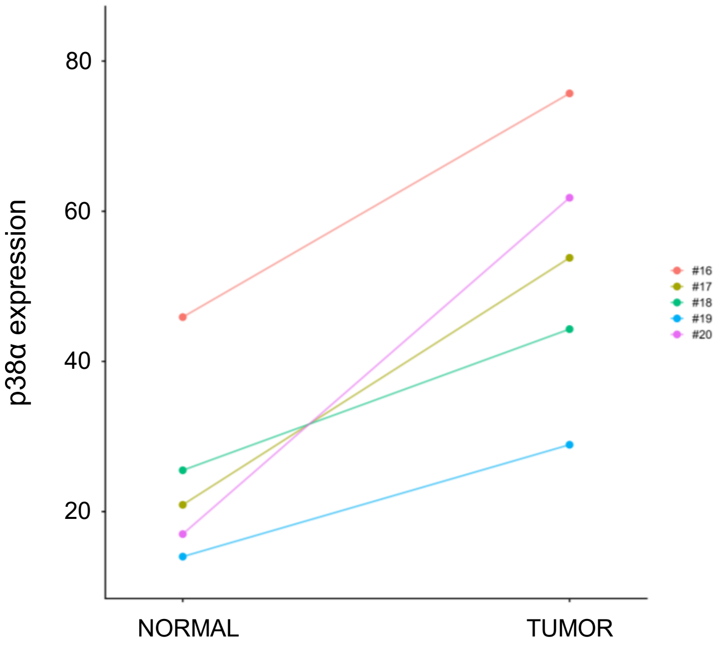

Supplement: Supplementary file 1 [file ijms-27-08550-s001.zip › ijms-4533951-supplementary.pdf]
